# Helical Plating Compared with Straight Plating and Nailing for Treatment of Proximal Third Humeral Shaft Fractures—A Biomechanical Study

**DOI:** 10.3390/medicina59112043

**Published:** 2023-11-20

**Authors:** Torsten Pastor, Ivan Zderic, Tatjana Pastor, Ludmil Drenchev, Hristo Kostov Skulev, Kenneth P. van Knegsel, Mark Lenz, Björn-Christian Link, Boyko Gueorguiev, Frank J. P. Beeres

**Affiliations:** 1 AO Research Institute Davos, 7270 Davos, Switzerland; torsten.pastor@luks.ch (T.P.); ivan.zderic@aofoundation.org (I.Z.); kenvanknegsel@hotmail.com (K.P.v.K.); 2 Department of Orthopaedic and Trauma Surgery, Lucerne Cantonal Hospital, 6000 Lucerne, Switzerland; bjoern-christian.link@luks.ch (B.-C.L.); frank.beeres@luks.ch (F.J.P.B.); 3 Department for Plastic and Hand Surgery, Inselspital University Hospital Bern, University of Bern, 3012 Bern, Switzerland; 4 Institute of Metal Science, Equipment and Technologies for Hydro- and Aerodynamics Center “Acad. A. Balevski”, Bulgarian Academy of Sciences, 1000 Sofia, Bulgaria; ljudmil.d@ims.bas.bg (L.D.); skulev@ims.bas.bg (H.K.S.); 5 Department of Trauma, Hand and Reconstructive Surgery, University Hospital Jena, 07747 Jena, Germany; mark.lenz@med.uni-jena.de

**Keywords:** unstable humeral fracture, helical plating, humerus, MIPO, biomechanics

## Abstract

*Background and Objectives*: The surgical treatment of proximal humeral shaft fractures usually considers application of either long straight plates or intramedullary nails. By being able to spare the rotator cuff and avoid the radial nerve distally, the implementation of helical plates might overcome the downsides of common fixation methods. The aims of the current study were (1) to explore the biomechanical competence of different plate designs and (2) to compare their performance versus the alternative treatment option of using intramedullary nails. *Materials and Methods*: Twenty-four artificial humeri were assigned to the following four groups for simulation of an unstable proximal humeral shaft fracture and instrumentation: Group 1 (Straight-PHILOS), Group 2 (MULTILOC-Nail), Group 3 (45°-Helical-PHILOS), and Group 4 (90°-Helical-PHILOS). All specimens underwent non-destructive, quasi-static biomechanical testing under loading in axial compression, torsion in internal/external rotation, and pure bending in four directions, accompanied by motion tracking. *Results*: Axial stiffness/displacement in Group 2 was significantly higher/smaller than in all other groups (*p* ≤ 0.010). Torsional displacement in Group 2 was significantly bigger than in all other groups (*p* ≤ 0.017). Significantly smaller coronal plane displacement was identified in Group 2 versus all other groups (*p* < 0.001) and in Group 4 versus Group 1 (*p* = 0.022). Significantly bigger sagittal plane displacement was detected in Group 4 versus all other groups (*p* ≤ 0.024) and in Group 1 versus Group 2 (*p* < 0.001). *Conclusions*: Intramedullary nails demonstrated higher axial stiffness and smaller axial interfragmentary movements compared with all investigated plate designs. However, they were associated with bigger torsional movements at the fracture site. Although 90°-helical plates revealed bigger interfragmentary movements in the sagittal plane, they demonstrated improved resistance against displacements in the coronal plane when compared with straight lateral plates. In addition, 45°-helical plates manifested similar biomechanical competence to straight plates and may be considered a valid alternative to the latter from a biomechanical standpoint.

## 1. Introduction

Humeral shaft fractures account for 1–3% of all human fractures [1,2]; however, their treatment can be a challenging endeavor. In some cases, conservative treatment is sufficient, although complication rates of up to 17% are reported [3]. In displaced shaft fractures of the proximal third, surgical treatment with different implants for nailing or plating is frequently chosen. Using minimally invasive plate osteosynthesis (MIPO), several studies reported such advantages as higher bone union rates versus intramedullary nailing and less neurological injuries versus open reduction and internal fixation (ORIF) [4,5]. However, higher injury rates of the axillary nerve are described when long straight plates are inserted with MIPO technique versus ORIF [6]. Moreover, the deltoid insertion is affected when straight plates are used [7]. A helical-shaped plate offers the potential to avoid distally the adjacent anatomical structures, such as the radial nerve. Several reports already demonstrated good clinical results using helical plates for treatment of humeral shaft fractures [8,9,10,11]. Furthermore, a recently published anatomical study demonstrated less axillary nerve stretching during insertion of a helically shaped PHILOS plate (DePuy Synthes, Zuchwil, Switzerland) with MIPO technique, as compared with straight PHILOS plates [12]. However, the helical plate design for fixation of proximal third humeral shaft fractures has not been subjected to a biomechanical investigation and compared to different osteosynthesis systems so far. Moreover, there is no consensus in the current literature on the optimal helical implant design, as there are reports considering 45°- to 90°-helical implants [8,9,11,13,14,15]. Therefore, the aims of this study were (1) to the biomechanical competence of different plate designs (straight, 45°-helical and 90°-helical) in an artificial bone model and (2) to compare their performance versus the alternative treatment option of using intramedullary nails.

## 2. Materials and Methods

### 2.1. Specimens and Study Groups

**Figure 1:**
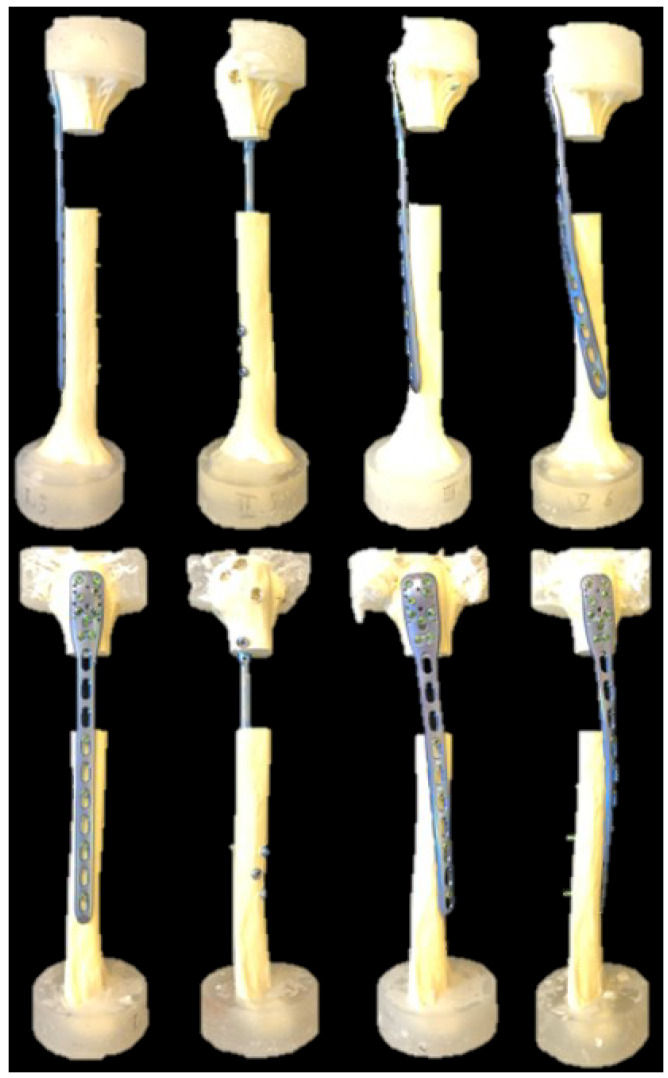
Specimens with a gap osteotomy simulating a right humerus fracture of the proximal third, visualized from left to right for Group 1 (Straight), Group 2 (Nail), Group 3 (45°-Helical) and Group 4 (90°-Helical) in anterior-posterior (**top**) and lateral (**bottom**) views.

Twenty-four right artificial humeri (5010, Synbone, Malans, Switzerland) made of polyurethane foam with an outer shell, simulating the inner cancellous core and the cortical bone of healthy individuals, were used. The specimens were assigned to four groups of six specimens each (*n* = 6) for implantation using either (1) a long 10-hole straight PHILOS plate (DePuy Synthes, Zuchwil, Switzerland) mounted on the lateral side of the humerus (Group 1 (Straight)), (2) a long intramedullary MULTILOC Humeral Nail (240 mm, Ø 7.0 mm, DePuy Synthes, Zuchwil, Switzerland) (Group 2 (Nail)), (3) a long 10-hole 45°-helical PHILOS plate mounted on the anterolateral side of the humerus (Group 3 (45°-Helical)), or (4) a long 10-hole 90°-helical PHILOS mounted on the anterior side of the humerus (Group 4 (90°-Helical)) (Figure 1).

### 2.2. Specimen Preparation/Surgical Technique 

All humeri were instrumented according to the guidelines of the implant manufacturer under fluoroscopic control (Siemens ARCADIS Varic, Siemens Medical Solutions AG, Erlangen, Germany). The PHILOS plates were prebent with the help of bending irons until the desired twisted rotation and fitted to the bone using a bending press. In the three groups with plated specimens, all nine holes of the proximal plate part (PHILOS rows A–E) were occupied with screws, reaching but not penetrating the second cortex. Distally, four screw holes (1, 3, 5, and 7, counted from distal) were occupied with bicortical angular stable self-tapping screws. The screw holes were chosen as close as possible to the gap osteotomy, as recommended by Stoffel et al. [16]. In Group 2 (Nail), the medullary canal of the specimens was opened and reamed (Ø 8 mm), all four proximal holes were occupied with 4.5 mm MULTILOC screws without additional screw-in-screw occupation, and a 5 mm endcap was used. The fifth proximal hole was occupied with a 3.5 mm ascending calcar screw, although this might not be routinely performed by some surgeons. Distally, all three holes were occupied with bicortical screws.

Following implantation, the proximal and distal 35 mm bone ends of all specimens were embedded in cylindrical polymethylmethacrylate (PMMA, SCS-Beracryl D28, Suter Kunststoffe AG, Fraubrunnen, Switzerland) forms. Thereby, the anatomical axis, defined as a straight line connecting the glenohumeral joint center and the cental aspect between the medial and lateral epicondyles at the elbow [17], was aligned with the axes of both embedding cylindric forms. Further, an AO/OTA 12-C3 fracture was simulated by means of two osteotomies 50 mm apart from each other. Symmetry in the fracture patterns was ensured by using a cutting jig. Optical markers were attached to the humeral head and shaft at a distance of 5 mm from the osteotomy gap for motion tracking, as described in previous work [18,19,20].

### 2.3. Test Setup 

**Figure 2:**
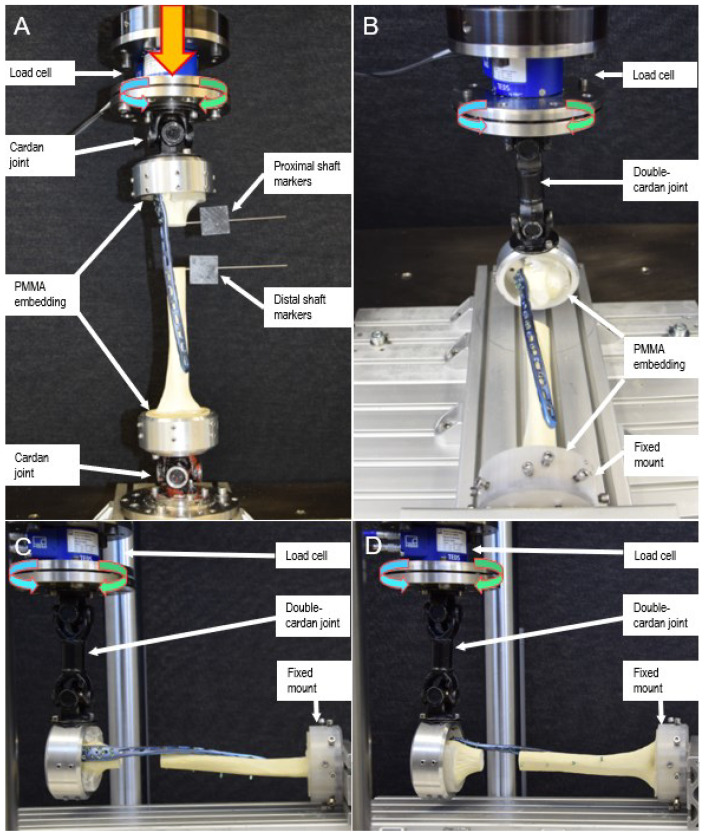
Setups with a 90°-helical plated specimen from Group 4 (90°-Helical) mounted for biomechanical testing. Arrows denote loading directions. (**A**): Setup with the specimen mounted for testing in axial compression and torsion in internal/external rotation, equipped with markers for motion tracking (Tests 1–3). (**B**,**C**): Setup with the specimen mounted for pure bending tests in the coronal plane (varus/valgus) (Tests 4, 5). (**D**): Setup with the specimen mounted for pure bending tests in the sagittal plane (flexion/extension) (Tests 6, 7).

A servo-hydraulic test system (Mini Bionix II 858, MTS Systems Corp., Eden Prairie, MN, USA), equipped with a 4 kN load cell, was used for biomechanical testing, implementing a test setup adopted from previous work [21] (Figure 2). Each specimen underwent seven non-destructive, quasi-static tests as follows. First, it was mounted between two cardan joints with the mechanical axis of the humerus in line with the machine axis, and axial compression was applied (Test 1). Second, using the same test setup, torsion in internal and external rotation was applied, simulating forces and moments generated during the external and internal rotation of the humerus, respectively, as induced by the rotator cuff (Tests 2, 3). Third, each specimen was mounted between a double cardan joint and a fixed basis to simulate pure bending. In this position, varus and valgus pure bending was first performed, simulating forces and moments induced to the humerus by the deltoid muscle and rotator cuff during abduction and adduction, respectively (Tests 4, 5). Further, each specimen was rotated 90° to perform flexion and extension pure bending (Tests 6, 7). During Tests 4–7, the double cardan’s rotational axis and the humeral shaft axis intersected each other at a 90° angle, allowing for transformation of the actuator torque to pure bending moments acting on the humerus while maintaining the axial force along the actuator axis at 0 N to keep the specimen free from shear stresses.

### 2.4. Loading Protocol

The loading protocol for Test 1—related to the axial movement of the machine actuator—consisted of three non-destructive quasi-static ramps from 0 N to 250 N at a rate of 0.1 mm/s, whereas the loading protocol for Tests 2–7—related to the torsional movement of the machine actuator—consisted of three non-destructive quasi-static ramps in both directions from 0 Nm to ±3 Nm at a rate of 1°/s. The first two ramps enhanced the specimen’s settling, whereas the third ramp was used for data evaluation.

### 2.5. Data Acquisition and Analysis

Machine data in terms of axial force, torque, and axial and rotational displacements were recorded from the machine controllers at 128 Hz [22,23]. Based on these data, axial stiffness and torsional stiffness in internal and external rotation, as well as varus, valgus, flexion, and extension stiffness, were calculated from the ascending slope of the load-displacement curve during the third quasi-static ramp of each corresponding test within the linear range between 0 and 250 N for axial compression or between 0 N and ±3 Nm for torsional and pure bending loading. Further, the coordinates of the markers attached to the specimens were acquired throughout the tests in axial compression and torsion in internal/external rotation (Tests 1–3) at 75 Hz using stereographic optical measurements applying contactless full-field deformation technology (Aramis SRX, GOM GmbH, Braunschweig, Germany). Based on these data, interfragmentary movements in terms of axial displacement—defined as the relative proximal to distal humeral shaft movement along the humeral shaft axis—, coronal plane displacement—defined as the relative angular proximal to distal humeral shaft bending movement in the coronal plane (varus/valgus)—, and sagittal plane displacement—defined as the relative angular proximal to distal humeral shaft bending movement in the sagittal plane (flexion/extension)—were assessed under 250 N axial compression. Shear displacement—defined as the relative proximal to distal humeral shaft movement in the fracture plane—and torsional displacement—defined as the relative angular proximal to distal humeral shaft torsional movement in the transverse plane—were assessed between −3 Nm and +3 Nm torsional loading.

### 2.6. Statistical Analysis 

SPSS software (IBM SPSS Statistics, V27, IBM, Armonk, NY, USA) was used for statistical analysis. Shapiro–Wilk test was applied to prove the normality of the data distribution. Significant differences between the groups with regard to the different types of stiffness, as well as regarding the axial displacement, coronal and sagittal plane displacements, and shear and torsional displacements, were detected with One-Way Analysis of Variance (ANOVA) and Bonferroni post hoc tests for multiple comparisons. Level of significance was set to 0.05. 

## 3. Results

**Figure 3:**
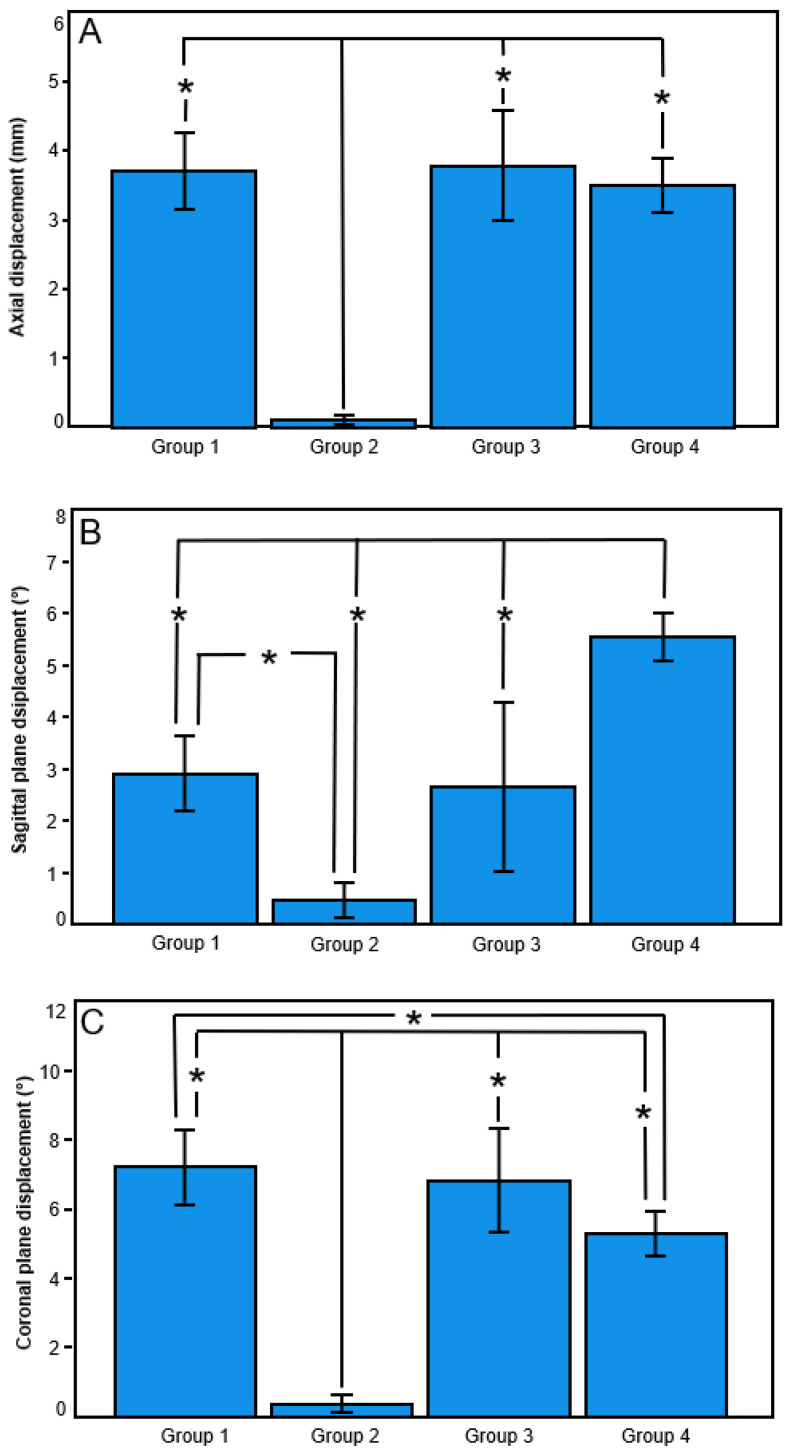
Outcome measures in Group 1 (Straight), Group 2 (Nail), Group 3 (45°-Helical) and Group 4 (90°-Helical) presented in terms of mean value and standard deviation for axial displacement (**A**), sagittal plane displacement (**B**), and coronal plane displacement (**C**) under axial loading. Stars indicate significant differences.

**Figure 4:**
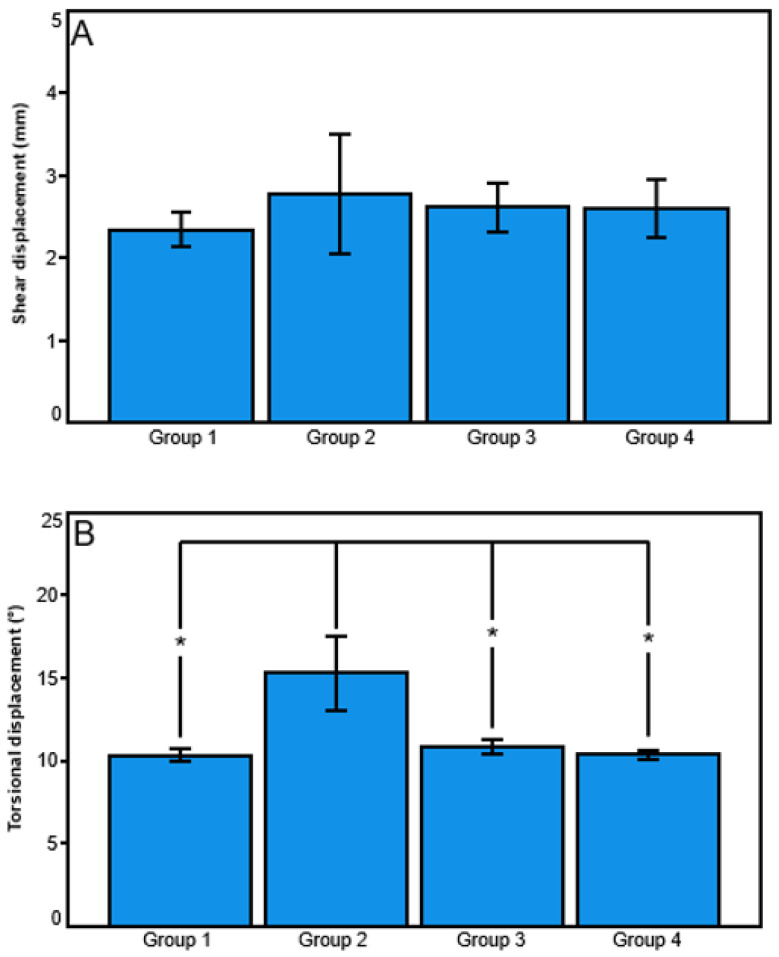
Outcome measures in Group 1 (Straight), Group 2 (Nail), Group 3 (45°-Helical) and Group 4 (90°-Helical) presented in terms of mean value and standard deviation for shear displacement (**A**) and torsional displacement (**B**) under internal and external torsional loading. Stars indicate significant differences.

The results from the current study are presented in Table 1 and Figure 3 and Figure 4. Axial stiffness—calculated from Test 1—was significantly higher in Group 2 (Nail) than in all other groups (*p* ≤ 0.010), with no further significant differences detected between the group pairs (*p* ≥ 0.541). Axial displacement—calculated from Test 1—was significantly smaller in Group 2 (Nail) versus all other groups (*p* < 0.001), with no further significant differences detected between the other group pairs (*p* ≥ 0.844). 

Varus stiffness—calculated from Test 4—was significantly higher in Group 2 (Nail) than in Group 1 (Straight) and Group 3 (45°-Helical) (*p* ≤ 0.013), with no further significant differences detected between the other group pairs (*p* ≥ 0.088). Valgus stiffness—calculated from Test 5—remained non-significantly different between the groups (*p* = 0.152).

Significantly smaller coronal plane displacement—calculated from Test 1—was detected in Group 2 (Nail) versus all other groups (*p* < 0.001) and in Group 4 (90°-Helical) versus Group 1 (Straight) (*p* = 0.022), with no further significant differences detected between the other group pairs (*p* ≥ 0.183). 

Flexion stiffness—calculated from Test 6—in Group 1 (Straight) was significantly higher compared with Group 2 (Nail) and Group 4 (90°-Helical) (*p* ≤ 0.031), with no further significant differences detected between the other group pairs (*p* ≥ 0.110). Extension stiffness—calculated from Test 7—was significantly higher in Group 1 (Straight) versus all other groups (*p* ≤ 0.015), with a trend toward significance in Group 2 (Nail) versus Group 4 (90°-Helical) (*p* = 0.057) and in Group 3 (45°-Helical) versus Group 4 (90°-Helical) (*p* = 0.059), and with no significant difference between Group 2 (Nail) and Group 3 (45°-Helical) (*p* = 0.989).

Significantly bigger sagittal plane displacement—calculated from Test 1—was detected in Group 4 (90°-Helical) versus all other groups (*p* ≤ 0.024), in Group 1 (Straight) versus Group 2 (Nail) (*p* < 0.001), and with a trend to significance in Group 3 (45°-Helical) versus Group 2 (Nail) (*p* = 0.075), with no significant difference between Group 1 (Straight) and Group 3 (45°-Helical) (*p* = 0.982). 

Torsional stiffness in internal and external rotation—calculated from Tests 2 and 3, respectively—remained non-significantly different between the groups (*p* ≥ 0.542). 

However, torsional displacement—calculated from Test 2 and Test 3—was significantly bigger in Group 2 (Nail) versus all other groups (*p* ≤ 0.017), with no further significant differences detected between the other group pairs (*p* ≥ 0.141). In contrast, shear displacement—calculated from Test 2 and Test 3—was not significantly different between the groups (*p* = 0.435).

## 4. Discussion

Although helical implants have been used for internal fixation as they can avoid the radial nerve in the distal humeral shaft [24], there is no consensus in the current literature about their optimal helical shape. The current study investigated the biomechanical competence—in terms of stiffness and displacements at the fracture site—of different plate designs and additionally compared them against the alternative treatment option of intramedullary nailing. The latter outperformed all investigated plate designs in terms of axial stiffness; however, these advantages became less prominent for bending stiffness, as well as for torsional stiffness in internal and external rotation, when compared with the plated constructs. This is in line with the observed smaller axial displacement, and coronal and sagittal plane displacements of the intramedullary nailed specimens compared with the plated ones during axial loading. However, significantly bigger torsional displacement was registered during torsional loading of the nailed specimens versus all plate designs. This biomechanical behavior can be explained with the toggling of the interlocking nail screws and underlines the importance of implementing optical motion tracking for evaluation of the interfragmentary movements at the fracture site—in agreement with previous findings highlighting the differences between nails and plates in a gap fracture model [25,26,27]. The reason for the higher axial stiffness of the nailed constructs is the force transfer which is located near the anatomical axis of the humeral shaft for intramedullary nailing in contrast to its more lateral location for plating. However, the toggling of the distal interlocking nail screws results in bigger torsional interfragmentary movements [28]. Therefore, different angular stable nails have been recently developed to address this problem, however, there is still an ongoing debate about these technical modifications, raising the question whether they could result in too stiff nailed constructs impeding bone healing.

Comparable results in terms of axial and torsional stiffness, as well as axial, torsional, and shear displacements, were found in the current study for all investigated plate designs. The fact that the upper extremity is mainly loaded in torsion has already been addressed in several reports highlighting the importance of the torsional stability of the implants used in this anatomical region [16,29]. However, especially in the early postoperative phase, when physical therapy is performed to prevent joint stiffness, high bending moments are applied to the upper extremity, that should not be neglected during biomechanical investigations. The current study registered bigger fracture gap movements in the coronal plane for straight versus 90°-helical plates, although no considerable differences were identified between them with regard to varus and valgus stiffness. In contrast, bigger fracture gap movements in the sagittal plane were found for 90°-helical plates compared with all other plate designs, with significantly higher flexion and extension stiffness for straight versus 90°-helical plates. An explanation for this phenomenon is the fact that during loading of the humerus in the sagittal plane, straight plates are stressed along their width dimension. In contrast, during loading of the humerus in the coronal plane, the straight plates are stressed along their thickness dimension, resulting in greater flexibility and bigger fracture gap movements. The 90°-helical plate demonstrates an opposing biomechanical behavior because during coronal plane loading it is stressed along the width dimension. Accordingly, this plate is stressed along its thickness dimension during sagittal plane loading. In contrast, the 45°-helical implants demonstrated a well-balanced behavior between stressing in the sagittal and coronal planes compared with straight and 90°-helical plates. 

Although the different implants used for plating of proximal humeral shaft fractures demonstrate variations in their biomechanical behavior, the reports on the implementation of helical plate designs in the range 45–90° are promising. The 45°-helical implants already revealed less radial nerve palsy versus straight plates, with comparable rates of uneventful healing [11,13]. Moreover, excellent clinical results were reported with the use of a 45°-helical plate [30]. Other authors published promising clinical outcomes when using 90°-helical implants [8,9,14,15]. In addition, anatomical studies have evaluated the feasibility and safety of applying helical plates with MIPO technique. Dissecting human cadaveric humeri after the application of a 90°-helical implant with MIPO technique, Gardner et al. identified the musculocutaneous nerve as the main structure being at risk during percutaneous screw insertion. With regard to the nerve location, the dangerous zone was determined as being at an average distance of 13.5 cm from the greater tuberosity [31]. Moreover, Dauwe et al. explored the axillary nerve stretching during insertion of a 90°-helical implant with MIPO technique versus straight plating. By evaluating the distance between the greater tuberosity and the plates, the authors reported a lower axillary nerve stretching and, hence, a lower risk of nerve damage with use of MIPO [12]. By contrasting the helical plate design, the recently introduced anatomical locking plate system (ALPS) (Zimmer Biomet, Warsaw, IN, USA) represents a different implant design to avoid the radial nerve—a 45° twisted plate with an additional anterior curvature. The difference between a twisted plate and a helical plate was emphasized by Fernandez [24]. The twisted plate winds around a single point in two distinct planes, whereas a helical plate follows a three-dimensional curve that lies on a cylinder while its angle progression remains constant in a plane perpendicular to the axis. The capability of the ALPS to avoid the axillary and musculocutaneous nerves has already been demonstrated in a cadaveric study with 10 specimens [32]. Furthermore, equitable healing rates and clinical outcomes—as compared with straight plates—were reported by Argyropoulos et al. [33]. However, despite the 45° twisted shape with an additional anterior curvature, the plate was not able to completely avoid the anterior part of the deltoid insertion, as it was partially compromised in all specimens after ALPS insertion with MIPO technique. Interestingly, Zamboni et al. evaluated the fit of helical and twisted plates on artificial and cadaveric bones and concluded that a 70° twisted shape fits best to the contour of the humerus, as compared with helical implants which lay more distant to the bone [34].

Despite the promising clinical reports, the optimal implant design has yet to be evaluated, as not only biomechanical aspects but also anatomical characteristics should be considered. A recent study demonstrated the safe feasibility of a 45°-helical plate inserted with MIPO technique [35]. Furthermore, neither the ALPS nor the 90°-helical plate was able to spare the deltoid insertion during MIPO. Therefore, it was concluded that, from an anatomical perspective, a 45°-helical implant offers the optimal shape as it can be pushed through the weaker central part of the deltoid insertion still avoiding the radial nerve distally. Furthermore, a recent biomechanical study demonstrated the inferior performance of a 90°-helical implant, as compared with straight plates during cyclic testing [35]. Additional human cadaveric biomechanical studies are needed to evaluate the competence of different plate designs, especially of the 45°-helical plate, via cyclic testing. Furthermore, future biomechanical research might investigate the application of helical plate design for fixation of femoral fractures, as an anatomical evaluation already demonstrated the safety and feasibility of its usage [36].

This study has some limitations inherent to all biomechanical investigations performed on artificial bones. First, only a limited number of twenty-four artificial humeri were used, restricting the generalization of the study findings; however, an appropriate study design was set to compare the biomechanical competence of the plate and nail designs. Second, an artificial bone model is incapable to simulate completely in vivo situations with surrounding soft tissues, swelling, and biological reactions following a bone fracture in a real human. Third, cyclic loading could not be performed due to the much weaker artificial bones—as compared with human cadaveric humeri. Fourth, when interpreting the results of the current study, one must keep in mind that a straight PHILOS plate was used, which was originally designed for lateral application. The helical plates were custom bent into shape; thus, their mechanical properties could have been affected throughout the bending procedure. An industrially produced implant might behave differently. Fifth, although plate bending was always performed by the first author, it could not be standardized. However, in a clinical setting, plate bending is also not standardizable and depends on the patient’s individual anatomy. Finally, intraoperative bending of the plates is time-consuming compared with the usage of straight plates. However, Wang et al. performed the bending procedure on a patient-specific three-dimensional print made from preoperative computed tomography data. With this technique, they were able to significantly reduce operation time and blood loss, as compared with conventional plate bending on artificial bones, as there was no need for corrections [15].

## 5. Conclusions

Intramedullary nails demonstrated higher axial stiffness and smaller axial interfragmentary movements compared with all investigated plate designs. However, despite similar torsional stiffness, they were associated with bigger torsional movements at the fracture site. Although 90°-helical plates revealed bigger interfragmentary movements in the sagittal plane, they demonstrated improved resistance against displacements in the coronal plane when compared with straight lateral plates. In addition, 45°-helical plates manifested similar biomechanical competence as straight plates and may be considered a valid alternative to the latter from a biomechanical perspective.

## Figures and Tables

**Table 1:** Parameters of interest in the study groups in terms of mean value and standard deviation.

Parameter of Interest	Group 1 (Straight)	Group 2 (Nail)	Group 3 (45°-Helical)	Group 4 (90°-Helical)
**Stiffness**				
Axial (Nm/mm)	66.4 ± 22.9	363.8 ± 133.2	74.8 ± 27.4	55.9 ± 19.2
Flexion (Nm/°)	0.84 ± 0.10	0.66 ± 0.07	0.72 ± 0.05	0.59 ± 0.14
Extension (Nm/°)	0.85 ± 0.14	0.69 ± 0.12	0.69 ± 0.09	0.56 ± 0.09
Varus (Nm/°)	0.66 ± 0.05	0.84 ± 0.12	0.65 ± 0.10	0.79 ± 0.07
Valgus (Nm/°)	0.65 ± 0.04	0.77 ± 0.19	0.64 ± 0.08	0.75 ± 0.07
Torsional–internal rotation (Nm/°)	0.32 ± 0.02	0.33 ± 0.05	0.31 ± 0.03	0.31 ± 0.04
Torsional–external rotation (Nm/°)	0.33 ± 0.02	0.34 ± 0.07	0.30 ± 0.02	0.31 ± 0.04
**Displacement under axial compression**				
Axial (mm)	3.71 ± 0.55	0.11 ± 0.06	3.79 ± 0.79	3.49 ± 0.39
Sagittal plane (°)	2.91 ± 0.72	0.48 ± 0.34	2.65 ± 1.62	5.53 ± 0.46
Coronal plane (°)	7.22 ± 1.08	0.37 ± 0.25	6.82 ± 1.49	5.28 ± 0.63
**Displacement under torsion in internal and external rotation**				
Shear (mm)	2.34 ± 0.20	2.77 ± 0.73	2.62 ± 0.30	2.60 ± 0.36
Torsional (°)	10.30 ± 0.38	15.31 ± 2.27	10.85 ± 0.42	10.35 ± 0.30

## Data Availability

The datasets used and/or analyzed during the current study are available from the corresponding author upon reasonable request.

## References

[1] Balfour G.W., Mooney V., Ashby M.E. (1982). Diaphyseal fractures of the humerus treated with a ready-made fracture brace. J. Bone Jt. Surg. Am..

[2] Mann R.J., Neal E.G. (1965). Fractures of the Shaft of the Humerus in Adults. South Med. J..

[3] Ali E., Griffiths D., Obi N., Tytherleigh-Strong G., Van Rensburg L. (2015). Nonoperative treatment of humeral shaft fractures revisited. J. Shoulder Elb. Surg..

[4] Zhao J.-G., Wang J., Meng X.-H., Zeng X.-T., Kan S.-L. (2017). Surgical interventions to treat humerus shaft fractures: A network meta-analysis of randomized controlled trials. PLoS ONE.

[5] Wen H., Zhu S., Li C., Chen Z., Yang H., Xu Y. (2019). Antegrade intramedullary nail versus plate fixation in the treatment of humeral shaft fractures: An update meta-analysis. Medicine.

[6] Robinson C.M., Khan L., Akhtar A., Whittaker R. (2007). The extended deltoid-splitting approach to the proximal humerus. J. Orthop. Trauma.

[7] Benninger E., Meier C. (2017). Minimally invasive lateral plate placement for metadiaphyseal fractures of the humerus and its implications for the distal deltoid insertion- it is not only about the radial nerve. A cadaveric study. Injury.

[8] Tan J.C.H., Kagda F.H.Y., Murphy D., Thambiah J.S., Khong K.S. (2012). Minimally invasive helical plating for shaft of humerus fractures: Technique and outcome. Open Orthop. J..

[9] Yang K.H. (2005). Helical plate fixation for treatment of comminuted fractures of the proximal and middle one-third of the humerus. Injury.

[10] Da Silva T., Rummel F., Knop C., Merkle T. (2021). Shoulder function after helical long PHILOS plate. Eur. J. Orthop. Surg. Traumatol..

[11] Da Silva T., Rummel F., Knop C., Merkle T. (2020). Comparing iatrogenic radial nerve lesions in humeral shaft fractures treated with helical or straight PHILOS plates: A 10-year retrospective cohort study of 62 cases. Arch. Orthop. Trauma Surg..

[12] Dauwe J., Grechenig P., Unterfrauner I., Schwarz A., Weiglein A., Hohenberger G. (2020). Axillary nerve elongation in humeral fracture plating: A cadaveric study for comparison between straight and helical Philos plates. J. Orthop..

[13] Arumilli B., Suhm N., Marcel J., Rikli D. (2014). Long PHILOS plate fixation in a series of humeral fractures. Eur. J. Orthop. Surg. Traumatol..

[14] Moon J.-G., Kwon H.-N., Biraris S., Shon W.-Y. (2014). Minimally invasive plate osteosynthesis using a helical plate for metadiaphyseal complex fractures of the proximal humerus. Orthopedics.

[15] Wang Q., Hu J., Guan J., Chen Y., Wang L. (2018). Proximal third humeral shaft fractures fixed with long helical PHILOS plates in elderly patients: Benefit of pre-contouring plates on a 3D-printed model-a retrospective study. J. Orthop. Surg. Res..

[16] Stoffel K., Dieter U., Stachowiak G., Gächter A., Kuster M.S. (2003). Biomechanical testing of the LCP--how can stability in locked internal fixators be controlled?. Injury.

[17] Anglin C., Wyss U.P. (2000). Review of arm motion analyses. Proc. Inst. Mech. Eng. H.

[18] Pastor T., Zderic I., Gehweiler D., Gardner M.J., Stoffel K., Richards G., Knobe M., Gueorguiev B. (2021). Biomechanical analysis of recently released cephalomedullary nails for trochanteric femoral fracture fixation in a human cadaveric model. Arch. Orthop. Trauma Surg..

[19] Pastor T., Zderic I., van Knegsel K.P., Beeres F.J.P., Migliorini F., Babst R., Nebelung S., Ganse B., Schoeneberg C., Gueorguiev B. (2023). Biomechanical analysis of helical versus straight plating of proximal third humeral shaft fractures. Arch. Orthop. Trauma Surg..

[20] Pastor T., Zderic I., Schopper C., Haefeli P.C., Kastner P., Souleiman F., Gueorguiev B., Knobe M. (2022). Impact of Anterior Malposition and Bone Cement Augmentation on the Fixation Strength of Cephalic Intramedullary Nail Head Elements. Medicina.

[21] Horn J., Gueorguiev B., Brianza S., Steen H., Schwieger K. (2011). Biomechanical evaluation of two-part surgical neck fractures of the humerus fixed by an angular stable locked intramedullary nail. J. Orthop. Trauma.

[22] Pastor T., Knobe M., van de Wall B.J.M., Rompen I.F., Zderic I., Visscher L., Link B.C., Babst R., Gueorguiev B., Beeres F.J.P. (2022). Low-profile dual mini-fragment plating of diaphyseal clavicle fractures. A biomechanical comparative testing. Clin. Biomech..

[23] Pastor T., Zderic I., Berk T., Souleiman F., Vögelin E., Beeres F.J., Gueorguiev B., Pastor T. (2023). New Generation of Superior Single Plating vs Low-Profile Dual Mini-Fragment Plating in Diaphyseal Clavicle Fractures. A Biomechanical Comparative Study. J. Shoulder Elb. Surg..

[24] Fernández Dell’Oca A.A. (2002). The principle of helical implants. Unusual ideas worth considering. Injury.

[25] Déjardin L.M., Guiot L.P., von Pfeil D.J.F. (2012). Interlocking nails and minimally invasive osteosynthesis. Vet. Clin. N. Am. Small Anim. Pract..

[26] Garlock A.N., Donovan J., LeCronier D.J., Houghtaling J., Burton S., Atkinson P.J. (2012). A modified intramedullary nail interlocking design yields improved stability for fatigue cycling in a canine femur fracture model. Proc. Inst. Mech. Eng. H.

[27] Nourisa J., Rouhi G. (2016). Biomechanical evaluation of intramedullary nail and bone plate for the fixation of distal metaphyseal fractures. J. Mech. Behav. Biomed. Mater..

[28] Flanagan B.P., LeCronier D., Kubacki M.R., Telehowski P., Atkinson P. (2014). A Method to Modify Angle-Stable Intramedullary Nail Construct Compliance. Iowa Orthop. J..

[29] Gautier E., Sommer C. (2003). Guidelines for the clinical application of the LCP. Injury.

[30] Zhang L., Chen L.-W., Zhang W.-J., Zhao C.-M., Huang B., Yu Q., Ni B. (2012). Treatment of proximal and middle one-third humeral fractures with lateral distal tibial helical plate. Eur. J. Orthop. Surg. Traumatol..

[31] Gardner M.J., Griffith M.H., Lorich D.G. (2005). Helical plating of the proximal humerus. Injury.

[32] Ekdahl M., Dominguez C., Pinedo M., López S., Gutiérrez V. (2021). New precontoured long locking plate for proximal metadiaphyseal fractures of the humerus: A cadaveric study for its use with the minimally invasive technique. JSES Int..

[33] Argyropoulos M., Kent M. (2018). Early Results of the A.L.P.S. Proximal Humerus Locking Plate. Open Orthop. J..

[34] Zamboni C., Carmo B.L., Moraes L.V.M., Hungria J.O.S., Mercadante M.T., Fucs P.M.M.B. (2019). A practical guide for the use of contour locking plates for the repair of humeral diaphyseal fractures with proximal extension. Injury.

[35] Pastor T., Kastner P., Souleiman F., Gehweiler D., Migliorini F., Link B.-C., Beeres F.J.P., Babst R., Nebelung S., Ganse B. (2022). Anatomical analysis of different helical plate designs for proximal humeral shaft fracture fixation. Eur. J. Trauma Emerg. Surg..

[36] Pastor T., Beeres F.J.P., Kastner P., Gehweiler D., Migliorini F., Nebelung S., Scaglioni M.F., Souleiman F., Link B.C., Babst R. (2022). Anatomical analysis of different helical plate designs for distal femoral fracture fixation. Injury.

